# Development of an Integrated Mariculture for the Collagen-Rich Sponge *Chondrosia reniformis*

**DOI:** 10.3390/md17010029

**Published:** 2019-01-05

**Authors:** Mert Gökalp, Tim Wijgerde, Antonio Sarà, Jasper M. de Goeij, Ronald Osinga

**Affiliations:** 1 Porifarma BV, Poelbos 3, 6718 HT Ede, The Netherlands; J.M.deGoeij@uva.nl (J.M.d.G.); ronald.osinga@wur.nl (R.O.); 2 Marine Animal Ecology, Wageningen University, P.O. Box 338, 6700 AH Wageningen, The Netherlands; tim.wijgerde@wur.nl; 3 Studio Associato Gaia, Piazza della Vittoria 15/23, 16121 Genova, Italy; a.sara@studioassociatogaia.com; 4 Department of Freshwater and Marine Ecology, Institute for Biodiversity and Ecosystem Dynamics, University of Amsterdam, P.O. Box 94248, 1090 GE Amsterdam, The Netherlands

**Keywords:** mariculture, sponge, *Chondrosia reniformis*, fishfarm, integrated multitrophic aquaculture

## Abstract

In this study, novel methods were tested to culture the collagen-rich sponge *Chondrosia reniformis* Nardo, 1847 (Demospongiae, Chondrosiida, Chondrosiidae) in the proximity of floating fish cages. In a trial series, survival and growth of cultured explants were monitored near a polluted fish farm and a pristine control site. Attachment methods, plate materials, and plate orientation were compared. In a first trial, chicken wire-covered polyvinyl chloride (PVC) was found to be the most suitable substrate for *C. reniformis* (100% survival). During a second trial, survival on chicken wire-covered PVC, after six months, was 79% and 63% for polluted and pristine environments, respectively. Net growth was obtained only on culture plates that were oriented away from direct sunlight (39% increase in six months), whereas sponges decreased in size when sun-exposed. Chicken wire caused pressure on explants and it resulted in unwanted epibiont growth and was therefore considered to be unsuitable for long-term culture. In a final trial, sponges were glued to PVC plates and cultured for 13 months oriented away from direct sunlight. Both survival and growth were higher at the polluted site (86% survival and 170% growth) than at the pristine site (39% survival and 79% growth). These results represent a first successful step towards production of sponge collagen in integrated aquacultures.

## 1. Introduction

The first attempts to farm sponges date back to the 19th century, presumably as a consequence of periodical depletion of “bath-sponge” stocks [1,2], or—in more recent times—in pursuit of a safer and economically more attractive alternative to wild collection [3,4]. Overfishing and repeated outbreaks of mass mortality events halted the ancient tradition of Mediterranean fishing of commercially important “bath sponge” species, such as *Spongia officinalis* (Linnaeus) and *Hippospongia communis* (Lamarck) [3,4,5,6]. Sponge mariculture has received increased attention over the last two decades (e.g., [7,8,9]; see also reviews or comparative studies by [10,11,12]), particularly driven by the discovery of biologically active metabolites in many sponges (e.g., [13,14]). Sponge mariculture could potentially provide for a sustainable supply of sponge-derived bioactive compounds and biomaterials.

Sponges can be co-cultured with other organisms in so-called integrated mariculture systems, in which sponges take up metabolic wastes from other system components, including bacterioplankton growing on these metabolic wastes [15,16,17,18,19]. This way, sponges can effectively reduce waste streams from fish farms [2,5,20], since they have been shown to exhibit retention efficiencies of up to 99% for nano- and picoplankton (e.g., [21,22,23]), while processing large volumes of water, up to 0.6 cm^3^ cm^−3^ sponge s^−1^ (e.g., [24,25,26]). Hence, a large-scale sponge culture facility that is constructed near a fish farm may positively affect the quality of the surrounding water. Conversely, the additional nutrition originating from the farmed fish may enhance the growth of the sponges in culture, thus providing a more efficient and profitable business.

In 2006–2007, an integrated mariculture approach using sponges was tested in the coastal waters around the Bodrum Peninsula, Turkey [27]. Two Mediterranean demosponge species with possible commercial interest, *Dysidea avara* (Schmidt, 1862) and *Chondrosia reniformis* Nardo, 1847 (Demospongiae, Chondrosiida, Chondrosiidae), were cultured at a pristine site (i.e., no fish farms within the nearest 30 km) and an organically polluted fish farm site, the latter sponges being directly cultured underneath an open cage fish farm. *D. avara* was chosen since it produces the bioactive compound avarol, a potential anti-psoriasis agent [14,28]. *Chondrosia reniformis* synthesizes large amounts of collagen, which is suitable for cosmetic and medical applications [29,30,31]. Type I & IV mammalian-like collagens can be effectively extracted from *C. reniformis* [32,33] and they can be used to promote the regeneration of human tissue and bone tissue engineering scaffolds [31]. *Chondrosia reniformis* showed better growth and survival rates at the pristine site, whereas *D. avara* grew and survived better at the polluted site [27]. The low survival rates of *C. reniformis* at the polluted site were largely due to the farming protocol used. *Chondrosia reniformis* is a highly plastic sponge, being able to de-attach and move around [34], a phenomenon that was frequently encountered using common culturing structures, such as pins, lines, plaques or metal/net grids [5,8,35,36]. To avoid displacement, explants of *C. reniformis* were put inside cages on the seafloor [27]. However, due to the high particle load in the water around the fish farm, the explants in these cages were suffocated by sediment.

This study describes progress towards the development of a raw collagen production pipeline using the sponge *C. reniformis* in an integrated multi-trophic aquaculture approach, i.e., by culturing the sponges in the vicinity of offshore floating fish cages. Using thin plastic plates as substratum, a series of consecutive trials were executed, aimed at developing an optimal, species-specific culture method. We monitored survival and growth rates of cultured explants of *C. reniformis,* thereby comparing a polluted fish farm site to a pristine site. Variables studied included methods for attaching explants to plates, plate materials and plate orientation. The culture methods (glue, cable-ties on plaques, net/mesh cover) were applied previously on other sponge species by several authors; for detailed information, see review by Duckworth et al. [10].

## 2. Materials and Methods

### 2.1. Mariculture Sites and Monitoring of Water Quality

**Figure 1:**
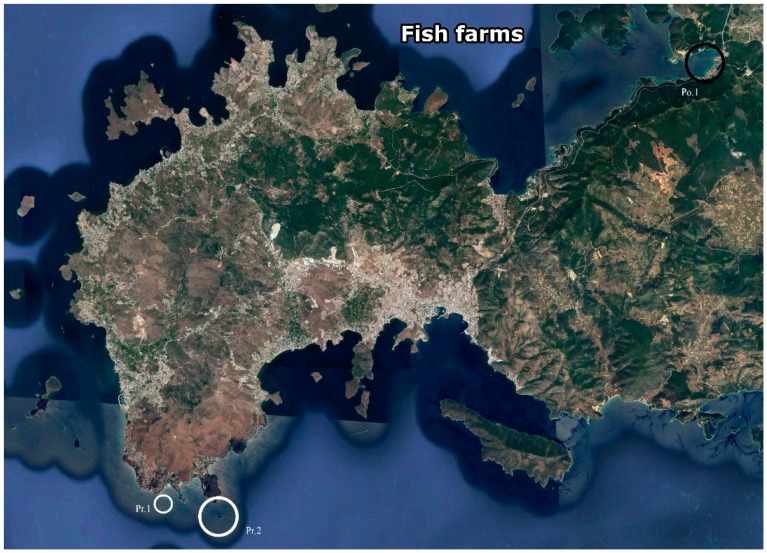
Map of the Bodrum Peninsula. Small white circle (Pr.1: Pre-culture site—Meteoroloji Bay). Large white circles (Pr.2: Pristine—Kargı island) and large black circles (Po.1: Polluted—Guvercinlik Bay) circles point the approximate locations of the sites used for growing sponge explants in this study. GPS coordinates 36.9444444, 27.27611111; 36.95166667, 27.30694444; 36.96861111, 27.45083333, respectively. (Source: Google Earth, 2018).

All of the studies were carried out in the coastal waters around the Bodrum Peninsula, Southwest Turkey (Figure 1). Meteoroloji Bay (Figure 1 Pr.1), a shallow area with an abundant population of *C. reniformis* was selected as a pre-culture and initial testing site (Trial 1). Based on water visibility (Secchi disk, cf. [37]) and organic loading (total organic carbon (TOC) measurements), two additional sites were selected for subsequent testing (Trials 2 and 3): Kargi Island (Figure 1 Pr. 2) at the Southern side of the Bodrum Peninsula was selected as a pristine site, whereas Guvercinlik Bay (Figure 1 Po. 1), located at the Northern side of the peninsula, was selected as a polluted site.

Water temperature (Uwatec Aladin Air X Nitrox dive computer, calibrated with mercury thermometer) and visibility were measured 17 times during periodic visits at the polluted site throughout the experimental period from April 2011 to December 2013. To determine organic loading, three replicate water samples (50 mL) were taken within 10 m from the culture platforms by SCUBA diving from each location for TOC analysis using the wet oxidation method [38]). TOC samples were stored in pre-combusted (450 °C for 6 h) 50 mL glass bottles with glass caps at −20 °C until analysis. Prior to analysis, sulphuric acid was added to the samples (end concentration 2 mmol L^−1^) to remove dissolved inorganic carbon species. The acidified samples were supplemented with sodium tetraborate and potassium persulphate and processed using segmented flow analysis (SFA) on a Continuous Flow Analyser (Skalar, Breda, The Netherlands). In SFA, TOC is first oxidized using UV light and then measured as CO_2_ while using infrared detection. The TOC detection limit of the method, as intercalibrated with other labs, is 25 μmol L^−1^, the average TOC variation among replicate measurements is 10 μmol L^−1^. The internal standards used were 3.3 μmol L^−1^ EDTA, 3.3 μmol L^−1^ of a humic acid, and 3.6 μmol L^−1^ phenylalanine.

### 2.2. Sponge Collection and Seeding

Sponge specimens were collected by SCUBA at 5–10 m water depth in the Bay of Meteoroloji (Figure 1 Pr.1). Explants were cut with sharp razor blades and detached from rock surfaces with a spatula [8,39], leaving the majority (at least 75%) of the donor sponge unaffected. The explants received maximally two cut surfaces and more than 50% of their surface was always covered with intact pinacoderm (i.e., the sponge outer tissue layer). The explant size ranged between 10–15 cm^2^ with an average thickness of 2 cm, and all of the explants had at least one osculum (i.e., outflow opening). Explants were stored in perforated plastic containers that allowed water flow and they were left underwater until the seeding operations started. To enable sponges to attach and acclimatize after seeding, the seeding plates with the explants were left horizontally next to the culture platforms for three days before the plates were secured to their spots on the culture frame [14].

### 2.3. Mariculture Trials

Within the period between April 2011 and December 2013, three subsequent mariculture trials were executed in order to develop and improve a culture method for *C. reniformis*.

1st mariculture trial, April 2011–June 2011: testing materials and attachment procedures—Sponge explants (*n* = 5 specimens per plate) were attached to four types of supports (autoclaved aerated concrete, white polyvinyl chloride (PVC), black PVC, and cemented PVC) using six different combinations of attachment methods and substrates (Table 1 (a)). The cementation of plates may improve attachment of the sponges to the support and enhance growth, since quartz and silica are found to promote collagen formation in sponges [34,40]. Accordingly, coarse sea-sand was used to make cement to cover the cemented-PVC supports. All of the supports were positioned in Meteoroloji Bay (Figure 1 Pr. 1) at 2–3 m water depth under overhangs or in crevices (i.e., not in direct sunlight) and fixed with diving weights.

**Figure 2:**
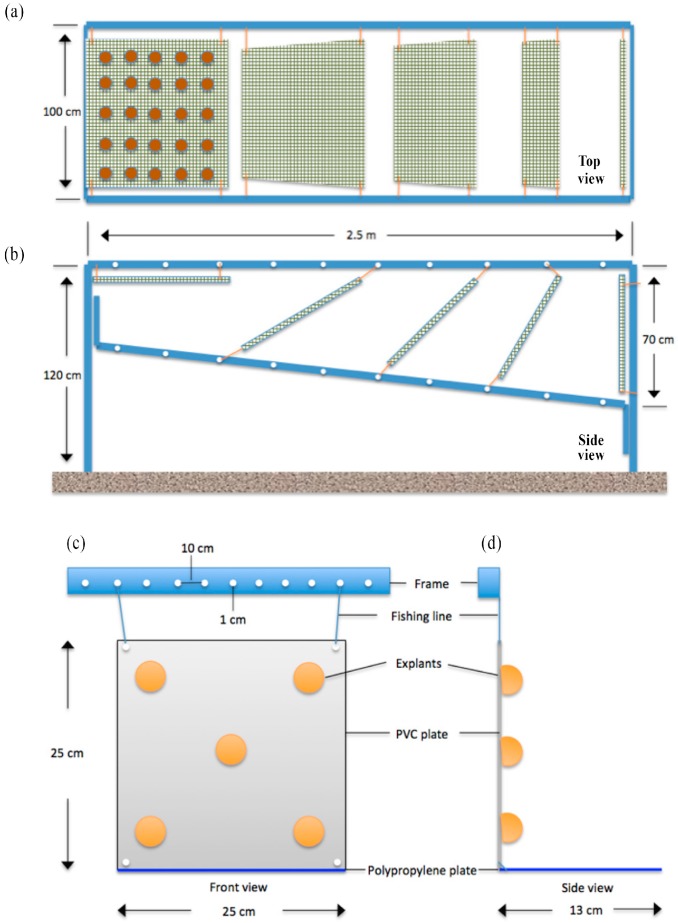
Schematic drawings of the culture platform designs. (**a**) 2nd trial—Top view; aluminum culture frame and attached 50 × 50 cm polyvinyl chloride (PVC) plates covered with chicken wire, each carrying 25 sponge explants on one side, totaling 50 sponges per plate. (**b**) 2nd trial—Side view; plates positioned at 9 different testing angles 0°, 30°, 45°, 60°, 90°, 120°, 135°, 150°, 180°. 3rd trial, (**c**) 3rd trial—front view, and (**d**) 3rd trial—side view showing positioning of glued *C. reniformis* explants on 25 × 25 cm PVC plates. PVC plates were secured tightly to the aluminum frame from four corners with 6-mm thick fishing lines, blue PP plates were attached to the bottom sides of the PVC plates.

2nd mariculture trial, June 2011–June 2012: testing culture plate orientation and site—Based on the results of the first trial, PVC plates were chosen for the second mariculture experiment. Explants (250 in total per site) were positioned on both sides of five 50 × 50 cm plates, with 25 explants on each side of every plate. In order to find the optimal positioning of the sponges, explants were cultured at nine different angles, under light (exposed side of plate) or dark (underside of plate) conditions, resulting in 10 different conditions (*n* = 1 plate per treatment) (0°, 30°, 45°, 60°, 90° light, 90°, 120°, 135°, 150°, and 180° dark; Supplementary Table S1, Figure 2a,b). In order to keep the sponges attached to the plates until natural attachment took place, the sponges were covered with chicken wire and left on the seabed for two days. After the attachment of the explants to the plates, the plates were mounted on a metal frame. Frames were installed in July 2011 at the two selected sites (Kargı Island (Figure 1 Pr. 2)—pristine and Guvercinlik Bay (Figure 1 Po. 1)—polluted) at a water depth of 10 m.

3rd mariculture trial, June 2012–July 2013: assessment of growth at the optimal culture orientation—Based upon the observations of the 2nd mariculture trial, it was decided to choose an angle of 90° for primary upscaling of the cultures. Two new frames were installed in early June 2012, one at the pristine site and one at the polluted site, each carrying 20 white PVC plates of 25 × 25 cm with a total of 100 explants of *C. reniformis* (5 per plate), attached using gel-based polyacrylate superglue. A gel-based polyacrylate superglue method was preferred over chicken wire to improve the handling time and reduce the weight and cost of the culture materials. In addition, horizontal blue polypropylene PP plates were placed underneath the 90° PVC plates to provide extra surface for the explants to attach and grow, should they fall (Figure 2c,d). Explants were grown for 13 months, photographed, and measured in June, July, August, September, October, and November 2012 (both sites), in March and May 2013 (polluted site only, due to weather restrictions), and in July 2013 (both sites).

### 2.4. Survival Rate Analysis and Sponge Explant Growth

Survival rates in Trial 1 were assessed by visual observation. For Trials 2 and 3, survival was monitored by underwater photography. Explants were photographed using a Nikon D300s digital single lens reflex camera (Nikon Corporation, Tokyo, Japan) and a Sigma 10–20 mm wide-angle lens set (Sigma Corporation, Ronkonkoma, NY, USA), coupled with dedicated Ikelite housing and an DS160 substrobe (Ikelite, Indianapolis, IN, USA). Survival was calculated from the initial and final number (N) of explants residing on the PVC and/or PP plates, as follows:

Survival = (N_final_/N_initial_) × 100 (1)

For Trial 3, as described above, blue PP plates were used to collect detached sponges. To calculate survival data, sponges that had fallen onto the PP plates were pooled with the sponges that remained on the PVC plates. However, fallen sponges were excluded from the growth analysis, as PP may affect sponge growth differently from PVC.

For Trial 2, explant growth rates were calculated by measuring wet weights by using a scale (Sinbo SKS 4514) at the start and end of the experiment. To reduce measurement error, the sponges were briefly wiped with clean paper to remove seawater for a duration of approximately 10 s. Growth was calculated from initial and final explant wet weights (WW) as follows:

Growth (%) = ((WW_final_ − WW_initial_)/WW_initial_) × 100 (2)

For Trial 3, growth was monitored by underwater photography using the same setup, as described above, for the monitoring of survival. Following each dive, recorded images were transferred to Photoshop CS5 software (Adobe Systems Incorporated, San Jose, CA, USA) and lens distortion was corrected using a Camera Raw 6.7.1 plug-in (Adobe Systems Incorporated, San Jose, CA, USA). The images were calibrated using known plate dimensions, peripheries of explants were marked, and surface area was calculated from pixel counts of the marked areas [39]. Growth was expressed as the increase in the number of pixels, calculated with the pixel counter function of the image editing software. At each time point, the growth in percentage increase in projected surface area was calculated from initial (at start of the time point) and final explant surface areas (A) as follows:

Growth (%) = (A_final_ − A_initial_)/A_initial_) × 100 (3)

To assess the correlation between surface area growth to both biomass and volumetric growth, an additional 20 sponges of random size were collected from a neighboring site. For all 20 sponges, the wet weights were determined, as described above. In addition, volume was determined by measuring displaced seawater in a graduated cylinder. Finally, surface area was determined by using photography and a ruler as a reference, and photographs were processed, as described above.

### 2.5. Statistical Analysis

The normality of data was tested by plotting the residuals of each dataset versus the predicted values, and by performing a Shapiro-Wilk test. Homogeneity of variances was determined using Levene’s test. All data were found to be normally distributed and showed homogeneity of variance after a log10 transformation (*p* > 0.05). Student’s independent *t*-test was used to determine growth differences between the light and dark group in the second trial, with *n* = 5 plates for both groups. A two-way mixed factorial ANOVA was used to determine the main and interactive effects of culture site and time on *C. reniformis* growth in the third trial, with culture site as a between-subjects factor, and time as a within-subjects factor. In all analyses, the culture plate was considered as an experimental unit, i.e., data of explants growing on a single plate was pooled. To correlate surface area to mass and volume, Pearson’s product-moment correlation was used. A *p*-value of less than 0.05 was considered to be statistically significant. Statistical analysis was performed with SPSS Statistics 22.0 (IBM, Somers, NY, USA). Graphs were plotted with SigmaPlot 12 (Systat software, San Jose, CA, USA).

## 3. Results

### 3.1. Polluted versus Pristine Site: Water Temperature, Visibility and TOC

**Figure 3:**
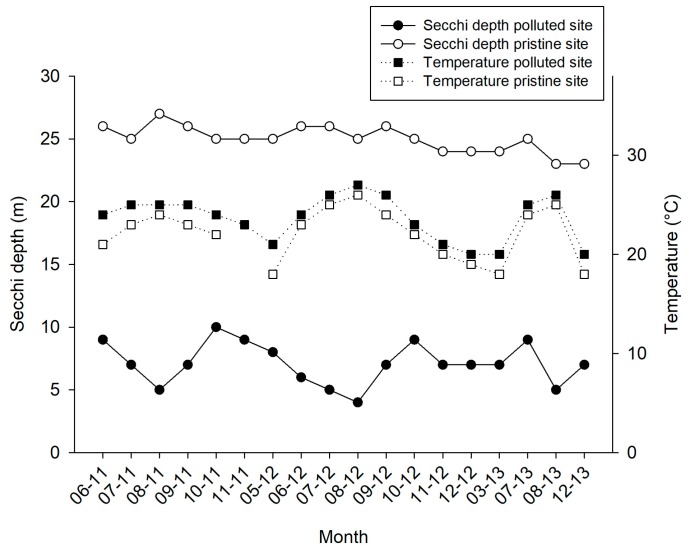
Water temperature (in °C, black squares with dottled line) and Secchi water depth (in m, black and white circles with continuous line) measurements for the pristine and polluted site over a 31-month time frame (June 2011–December 2013).

Visibility was on average 3.8 times lower at the polluted site (6.5 ± 1.7 m; mean ± SD throughout text unless stated otherwise) when compared with pristine site (25 ± 1.1 m; Figure 3). The water temperatures that were recorded at the polluted site ranged between 19–26 °C (Figure 3). During summer periods, especially in August, as a result of intensive fish feeding activities, Secchi disk water depth dropped to 4–6 m at the polluted site (Figure 3). TOC levels at the polluted site (280 ± 0.07 µmol L^−1^) were 2.4 times higher than the TOC levels at the pristine site (110 ± 0.01 µmol L^−1^).

### 3.2. 1st Mariculture Trial: Testing Materials and Attachment Procedures

The air concrete material was found to be unsuitable for further experimentation, as none of the explants attached to it (Table 1 (a)). In addition, the material was positively buoyant in seawater, which hampered easy handling. There was no difference in preference between white and black PVC (80% survival for both plates), as sponges attached equally well to both substrates without showing any signs of disparity (Supplementary Figure S1a,b, Table 1 (a)). Cable-ties gave a better recovery percentage than super glue (80% vs. 60%), but, in addition to increasing handling time, cable-ties also triggered the dispersion of *C. renifomis* explants into two parts for both black and white PVC’s (fission; Supplementary Figure S1b). The combinations PVC/chicken wire and cemented PVC/cable-tie were the most successful methods in terms of recovery percentage (all sponges survived on plate). However, the cost of material, plate weight, and handling time were factors favoring the PVC/chicken wire method (Table 1 (a)). Accordingly, PVC/chicken wire method was selected for the 2nd trial.

### 3.3. 2nd Mariculture Trial: Testing Culture Plate Orientation and Site

**Figure 4:**
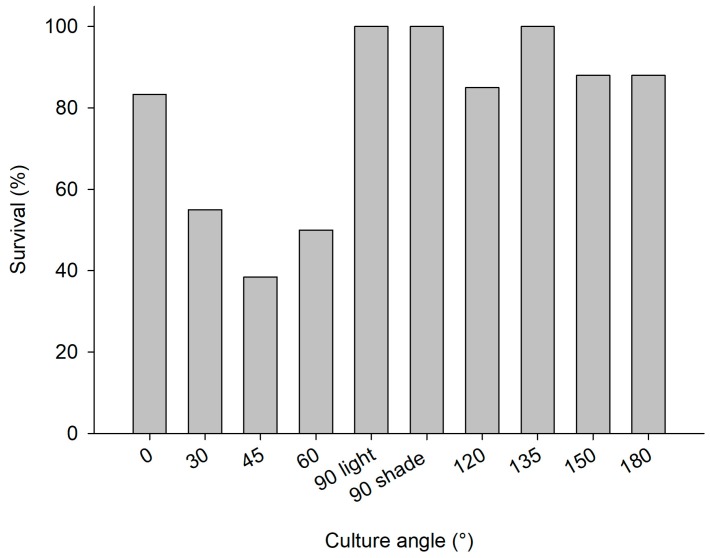
2nd mariculture trial, polluted site, June 2011–June 2012; survival percentage of *C. reniformis* explants on PVC plates with various angles (0–90° light represents PVC plates with greater light exposure and 90° shade −180° plates receiving less light exposure).

**Figure 5:**
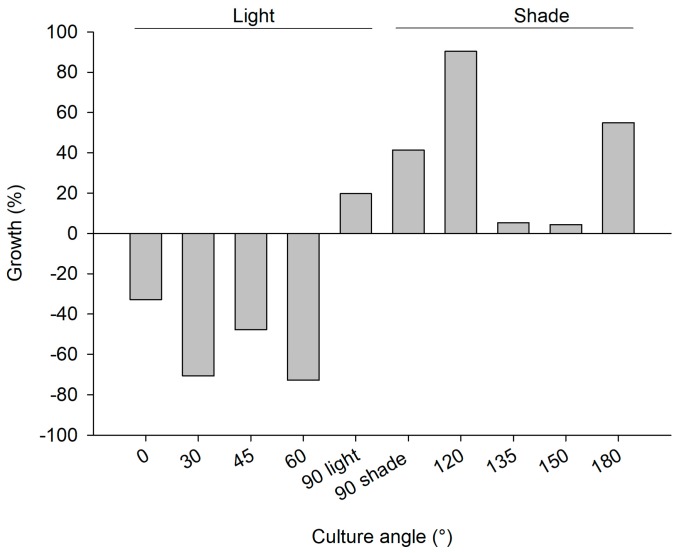
2nd mariculture trial, polluted site June 2011–June 2012; growth rate as percentage wet weight increase of *C. reniformis* explants on PVC plates with various angles (0–90 light represents PVC plates with greater light exposure and 90 shade −180 plates receiving less light exposure; 25 explants for each plate).

The explants at the polluted site (Supplementary Figure S1c) and pristine site (Supplementary Figure S1d) showed signs of bacterial infections and decay within a week after initiation of the cultures, causing initial losses at both sites (4.8% and 2% of explants deteriorated in the polluted and pristine sites, respectively). Among the explants that survived the initial deterioration, overall survival after six months was slightly better at the polluted site (79% of 238 explants survived at the polluted site and 63% of 245 explants survived at the pristine site; Table 1 (b)). The culture frame at the pristine site was found to be demolished, when revisited in May 2012. It was found 50 m away from the culture site. As a consequence, it was not possible to deduce annual survival and growth rates for the culture at the pristine site. At the polluted site, survival was highest among sponges that were put at an angle of 90° or higher (Figure 4). Growth rates were highly variable among treatments (Figure 5), but the average growth at “light” angles of 0–90° (−41 ± 38%; negative values points to loss of WW biomass) was significantly lower than the average growth at “shade” angles of 90–180° (39 ± 36%; Student’s *t*-Test *z* = −3.4, *p* < 0.01, *n* = 5). The 90° plate was selected as the preferred culture orientation in Trial 3, based on the survival rate and the ease of operation (photography, measurements, and handling; see Table 1 (b) for details). Photographic measurement of growth was found to be impossible with the PVC-chicken wire method as a result of continuous movement, splitting, and fusing of *C. reniformis* explants, and epibiont growth. In addition, chicken wire compressed the explants, which may not be beneficial for their development. Also, installing the large 50 × 50 cm PVC plates was time consuming. Therefore, smaller (25 × 25 cm) PVC plates were used in Trial 3 and superglue was selected as the attachment method.

### 3.4. 3rd Mariculture Trial: Assessment of Sponge Culture Productivity Polluted vs. Pristine Site

**Figure 6:**
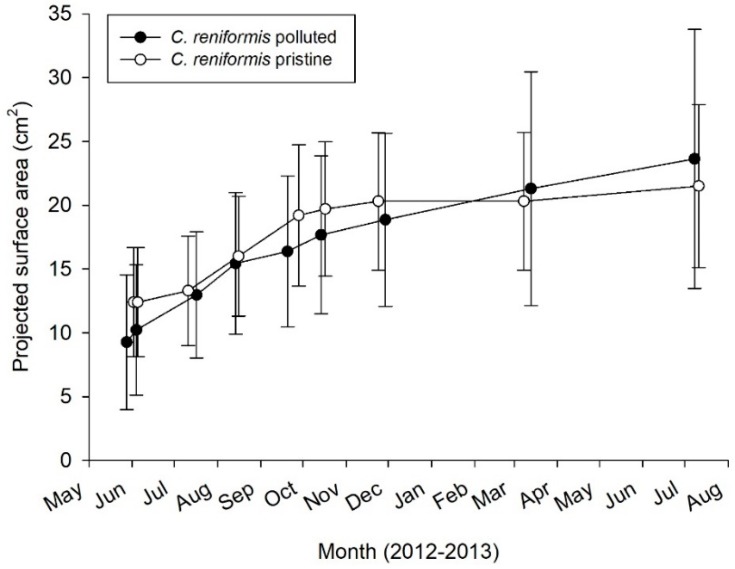
3rd mariculture trial. Annual growth rate as surface area increase for *C. reniformis* (*n* = 15–16 plates) explants in polluted and pristine sites.

During the first week of the experiment, 69 (polluted) and 70 (pristine) out of 200 explants dropped off the PVC plates. Fortunately, the PP plates that were placed under the PVC plates were able to catch 55 (at polluted site) and nine (at pristine site) of these explants, which attached onto the PP plates and continued to increase their surface area. Because they could not be related anymore to their original size, explants that were attached on the PP plates were left out of the surface area increase analysis. However, they were included in calculation of survival rates, which were highly different between the pristine and polluted sites (39–86%, respectively—Table 1 (c)). A total of 61 explants survived on the vertical PVC plates (30 explants on 15 plates at the pristine site, 31 explants on 16 plates at the polluted site), which were used for surface area increase analysis. The average increase in surface area over time of these *C. reniformis* explants is presented in Figure 6. After being cultured for 13 months, the average surface area increase was 79.0 ± 37.4% at the pristine site and 170.4 ± 109.1% at the polluted site (Table 1 (c)). Both culture site and time had a significant main effect on sponge surface area increase rates (Table 2). At both sites, explant surface area increased significantly, but it slowed down after the first six months at both sites (two-way factorial ANOVA, F_1,27_ = 55.550; *p* < 0.001), and for the pristine site even stalled after six months. Surface increase was significantly higher at the polluted site as compared to the pristine site (two-way factorial ANOVA, F_1,27_ = 14.439; *p* = 0.001), irrespective of time.

For *C. reniformis*, a highly significant correlation between surface area and wet weight was found (Pearson correlation, *r* = 0.92, *n* = 20, *p* = 0.000, two-tailed, Supplementary Figure S2), as well as between surface area and volume (*r* = 0.92, *n* = 20, *p* = 0.000, two-tailed, Supplementary Figure S3). The relationships are size-independent, leading to fixed conversion factors of 1.2 g wet mass per cm^2^ of surface area and 1.1 cm^3^ sponge volume per cm^2^ surface area, respectively.

## 4. Discussion

This study explored the feasibility to integrate fish culture with a biomedically promising Mediterranean sponge species, *C. reniformis*. The main aim of the study was to derive the best mariculture practices of *C. reniformis* from a series of subsequent culture trials.

### 4.1. Explant Survival Rates

Survival of explants can be compromised by detachment and by disease. In terms of initial survival, cable-ties and chicken wire were the most effective means of attaching explants onto PVC substrates, with glue giving a slightly lower survival. In the long term, however, the use of chicken wire (mesh culture) gave ambiguous results. Sandwiched mesh structures were designed to promote the explants to grow out of the pocket and to ease harvesting [8]. Mesh culture that is used in turbid waters might reduce water flow and subsequently decrease available food for the explants if mesh size is too small [10]. Although the mesh size used in the second trial was sufficiently large (5 × 5 cm) as recommended in [41], after some time the space between meshes and the PVC plate was covered by epibionts, and the mesh did not prevent some explants from moving or even dropping themselves off the plate. Despite these drawbacks, the survival rate at the polluted site after one year was 79%, which is higher than in the study by [8], who reported 55% survival after seven months and who lost entire *C. reniformis* explants with the sandwiched mesh method. However, the mesh method is labor intensive, especially when considering that increased cleaning of biofouling on the mesh is recommended. By attaching the explants with glue in the third trial, it was anticipated to reduce both handling time and fouling. Despite the predicted improvements regarding initiation time (May vs. June) and culture angle (all at 90°), the third trial showed low survival for the pristine site. This was probably due to occasional strong currents that prevail at this site, which may make the explants more prone to dropping of the plates and physical removal from the site. During the whole month of September 2013, flow velocities above 20 cm/s were recorded at this site by analyzing the velocity of neutrally buoyant particles (video clips of laterally moving natural particles, data not shown). *Chondrosia reniformis* inhabits both nearly stagnant to occasional high flow waters (M. Gokalp; personal observation), however the attachment of explants to PVC plates is probably less firm than attachment to natural substrates, especially during the acclimatization time after wounding them to explant the parent sponges. At the polluted site, the use of glue instead of chicken wire did slightly improve long-term survival rate, which shows that gluing is a suitable method to attach explants of *C. reniformis*. It is also the fastest and easiest method. A future recommendation is to perform the initial acclimatization (of 7–10 days; see [42]) at a more secluded site, after which the attached explants are placed at the study site.

During culture Trial 2, initial sponge survival was compromised by disease-like phenomena. Bacterial infections, which were possibly due to late seeding of explants in Mid-June with relatively higher water temperatures, might have been responsible for the initial losses at both sites. High water temperatures in summer have been reported to be a risk for sponge mariculture in temperate and subtropical climates [11,39,41,43], as it makes cuttings more vulnerable to bacterial attack, although such increased vulnerability had not been observed in our earlier studies on this species in this area [27].

Culture angle directly affected explant survival, mainly in association with prevailing light levels. Lower light levels at the more turbid polluted site may, therefore, also explain the higher explant survival at the light-exposed angles at the polluted site. These results corroborate the findings of [35], which purport *C. reniformis* prefers shaded habitats.

### 4.2. Explant Growth

Since surface area of *C. reniformis* showed a size-independent relationship with wet weight and volume, surface area can be used as a proxy for growth. This enables a direct comparison of growth data obtained for this species using different methods.

Culture of *C. reniformis* has been considered to be difficult, to even unsuitable with the methods applied [5,8]. Wilkinson and Vacelet [35] reported moderate growth rates of 95% per year (55 weeks doubling time in volume, measured using volume displacement) when *C. reniformis* was cultured under shaded conditions. Ref. [27] obtained grow rates of 100 to 200% per year when growing *C. reniformis* on the bottom of metal wire cages under pristine conditions, but this study failed to achieve such results at a fish farm site as the explants cultured were smothered by effluents from the fish farm. Conversely, the current study demonstrates that if cultured using an appropriate method, *C. reniformis* will survive and grow (up to 170% in 13 months), even in a fish farm environment with a considerable particle load. These growth rates are considerably higher than those reported for naturally growing specimen. Garrabou and Zabala [36] reported an in situ growth rate of 2.3% per year (deduced from two-dimensional (2D) areal growth) for *C. reniformis*, which was an order of magnitude lower than the growth rate of three other Mediterranean sponge species in their study *Hemimycale Ccolumella* (Bowerbank)*, Oscarella lobularis,* and *Crambe Crambe* (Schmidt). They ascribed the slow growth rate of *C. reniformis* to a greater energy investment in tissue production per unit area as a result of its thick collagenous cortex. However, the data found by Osinga et al. [27] and those from the current study indicate that in aquaculture, *C. reniformis* exhibits growth rates that are nearly two orders of magnitude higher than the in situ rates reported by Garrabou and Zabala [35]. Under optimal circumstances, the production of collagen is apparently not hampered by energy input. The current results show a clear potential for collagen production through the aquaculture of *C. reniformis*. The highly variable growth of *C. reniformis* under different conditions and the high variability within treatments highlight the need for further optimization studies.

During Trial 3, *C. reniformis* surface area increase rates were significantly different between culture sites, with an approximate two-fold higher growth at the polluted site. This may relate to the higher food availability—i.e., higher TOC concentration as a result of fish farm activities—and, as mentioned earlier, correspondingly lower light levels at the polluted culture site. Hence, the combination with fish farming is potentially beneficial for culture success of this sponge species. The surface area increase of *C. reniformis* was clearly higher in the first six months after initiation of the cultures, regardless of culture site. Although this may partially be explained by seasonal effects (growth might cease in autumn and winter [11,44]), it is possible that the sponges exhibit lower specific growth rates when being in culture for a longer period [39]. This could be due to initial enhanced surface area increase due to explant cutting [45], which could hamper growth at later stages, due to high costs of wound healing and regeneration [42]. Fast initial surface area increase was also found in a side experiment where the explants were cultured starting in autumn 2013 (data not shown), pointing towards a wound healing and regeneration effect rather than a seasonal effect, but this observation needs to be further investigated.

### 4.3. Culture of Chondrosia reniformis—Best Practices

As stated in Schippers et al. [11], initial mariculture trials should span a complete annual cycle in order to perceive effects of seasonality, substrate preference, and growth physiology of the sponge, and possible external impacts to the culture site, such as the occurrence of fouling and specific sponge predators, boat traffic and anchoring, and the presence of fishermen and divers. Accordingly, in this study, valuable information was acquired regarding the preferences for attachment, survival, and growth of *C. reniformis* during the first two trials. *Chondrosia reniformis* explants attached to PVC plates tend to move on the plate, thus obfuscating multiple genotypic comparisons on one plate (our study was initially designed to investigate genotype effects, but this part of the study could not be completed due to random movement of the explants over the plates). In addition, fusion and fission of explants makes the proper assessment of survival difficult. Even though attachment to the PVC plates has succeeded, some *C. reniformis* still found ways to divide their body into several parts, moved around the PVC plate (possibly in pursuit of shaded areas), or dropped themselves to the ground possibly in search for better living conditions. Survival and growth is best at culture angles of 90° and above, where explants are not being exposed to direct sunlight, as *C. reniformis* performs better at low illumination levels.

In experiments 2 & 3, the initial losses and/or droppings of explants were slightly high and unpredictable, despite the variety of methods applied. Once attached for a longer time, the explants would remain attached. Therefore, initial losses and/or droppings are the main problem to be solved to secure better culture performance. Restraining bacterial attack on freshly cut explants by initiation of cultures early in the season (spring) and preventing exposure to high currents during the first months should be practiced together with the best performing methods regarding attachment.

Based upon the three mariculture trials described above, the following best practices have been deduced for culturing *C. reniformis* in sea-based aquaculture under turbid conditions:

1. Culture method: Sponge explants cut from parent sponges are glued to PVC plates using gel-based polyacrylate superglue. PVC plates are best positioned vertically onto frames and they should be extended with a basket on the bottom site to recover explants falling off the plates. Chicken wire may be applied during the first few weeks after explanting to prevent early losses but should be removed once the attachment is stable. Prolonged use of chicken wire cover tends to hold sediments and promotes epibiont growth and hence undesired space competition with the cultured sponge.
2. Site selection: Sites should not be prone to strong fluctuations in weather. The area should be secured and should be clear of boat traffic and anchoring [10,11]. Sites should be carefully assessed for (e.g., seasonal) strong currents. High water turbidity and increased load of organic content associated with the presence of fish farms does not appear to hamper growth of *C. reniformis* on vertical plates, making this sponge an interesting candidate for integrated multitrophic mariculture. Daily fish feeding activities and occasional net replacing hinders the use of culture platforms inside the fish farm area. Thus, sponge culture platforms have to be placed outside boat traffic area. To eliminate this problem, one method that we consider for future applications is using layered scallop lanterns placed in between an anchor and a submerged buoy system (just outside the fish farm culture area), a method that was successfully applied by both Duckworth et al. [46] and Kelly et al. [12], for *Latrunculia wellingtonensis*, *Polymastia croceus,* and (*Heterofibria*) *manipulatus*, respectively.
3. Seasonality: Initiating a culture of *C. reniformis* in the Mediterranean is best done in either spring (April-May) or autumn (October-November) to prevent bacterial infections following cutting of explants from parent sponges.

### 4.4. Recommendations for Future Research

Culture success can be further improved by optimizing the period of culture. Optimal culture time can be determined by observing sponge growth rates over a period of two or more subsequent years. Page et al. [39], found reduced growth rates for *Mycale (Carmia) hentscheli* (Bergquist & Fromont) [47] over time. The growth rates in their study dropped from 2437% year^−1^ to 1355% year^−1^ from the first to the third culture period. Moreover, the growth rates of cloned sponges harvested from cultured explants should also be followed, as Page et al. [39], found reduced growth rates and even negative growth through repeated cloning (F0 to F2).

Other important aspects to include in future studies are seasonality (e.g., is the fast initial growth observed in this study season-influenced or a wound-healing response that is irrespective of season) and genotypic variability.

## Figures and Tables

**Table:** 

**a. 1st trial** Material test and attachment procedures	**Material**	**Attachment Method**	**Advantage**	**Disadvantage**	**Result**
	Air-concrete	Iron screw		No attachment	Not suitable
	PVC—white	Cable-ties	Survival (80%)	Dispersion of explants	Not selected for 2nd trial
		Superglue	Ease of operation	Lower survival (60%)	Selected for 3rd trial
		Chicken wire	High survival (100%)	handling time	Selected for 2nd trial
	PVC—black	Cable-ties	Survival (80%)	Dispersion of explants	No preference; the color of the plate did not affect the result
	Cemented PVC	Cable-ties	High survival (100%)	Cost, handling time and weight	Not selected

**Table:** 

**b. 2nd trial** Testing culture orientation and site	**Site**	**Material**	**Disadvantage**	**Survival Rate**	**Average Growth**	**Result**	**Orientation (°)**
	Pristine	PVC chicken wire	Squeezed explants, resulted in unwanted epibiont growth, time & cost	63% survival after 6 months of culture	Culture frame demolished by an anchor	Chicken wire method is not suitable	90° was selected for the next trial
	Polluted			79% survival after 6 months & 1 year of culture	39.2 ± 36.2% in 12 months for dark angles		
					−40.9 ± 37.7% in 12 months for light angles		

**Table:** 

**c. 3rd trial** Assessment of productivity at the optimal culture orientation	**Site**	**Species**	**Survival Rate**	**Average Growth**	**Growth per Culture Interval**		**Range of Growth for Individuals**
					**0–6 Months**	**7–13 Months**	
	Pristine	*C. reniformis* (*n* = 15 plates)	39%	79.0 ± 37.4% in 13 months	69.8 ± 33.6%	5.4 ± 5.7%	−3.6–135.6%
	Polluted	*C. reniformis* (*n* = 16 plates)	86%	170.4 ± 109.1% in 13 months	114.0 ± 94.6%	30.1 ± 27.9%	0.8–322.9%

**Table 2:** Two-way mixed factorial ANOVA, demonstrating main and interactive effects of culture site and time on *C. reniformis* growth rates (*n* = 15–16).

Factor	F	df	Error	*p*
Culture site	14.439	1	27	0.001 **
Time	55.550	1	27	0.000 **
Culture site x Time	2.686	1	27	0.113

** Indicates significant effect (*p* < 0.01).

## Supplementary Materials

The following are available online at http://www.mdpi.com/1660-3397/17/1/29/s1, Figure S1: Overview of culture methods; (a) 1st trial; explants tie-wrapped to black PVC, experiment start (b) 1st trial; sponges tie-wrapped to white PVC, 1 year later explants splitting into two fragments and relocating position. (c) 2nd trial, polluted site; image of 50 × 50 cm plates, sponge explants secured with chicken wire (0–30° plates can be seen, 25 additional explants are on the other side of each plate). (d) 2nd trial, pristine site; infected explants shortly after seeding. (e) 3rd trial, polluted site; first upscaling of aquaculture of Chondrosia reniformis on vertical PVC plates. Explants are attached with gel-based polyacrylate superglue to PVC plates (10 plates per site). (f) 3rd trial, pristine site; explants after 4 months of culture. Some of the explants tend to travel towards the polypropylene plate and grow on it, but others prefer to stay on the PVC plates. Figure S2: Correlation between surface area (cm^2^) and wet weight (g) for Chondrosia reniformis (Pearson correlation, *r* = 0.92, *n* = 20, *p* = 0.000, two-tailed). Figure S3: Correlation between surface area (cm^2^) and volume (mL) for Chondrosia reniformis (Pearson correlation, *r* = 0.92, *n* = 20, *p* = 0.000, two-tailed). Table S1: Experimental design of aquaculture trials 2 and 3. In Trial 3, 10 explants were taken per parent sponge, five explants were attached to each plate.
