# Supplementary material for: Development of an Integrated Mariculture for the Collagen-Rich Sponge Chondrosia reniformis
Source: Mar Drugs. 2019 Jan 5;17(1):29. doi: 10.3390/md17010029 (PMC6356691; doi:10.3390/md17010029)
Supplement: Supplementary file 1 [file marinedrugs-17-00029-s001.pdf]

marine drugs

Supplementary Materials

Development of an Integrated Mariculture for the Collagen-Rich Sponge *Chondrosia reniformis*

Mert Gökalp [1,2,*], Tim Wijgerde [2], Antonio Sarà [3], Jasper M. de Goeij [1,4] and Ronald Osinga [1,2]

[1] Porifarma BV, Poelbos 3, 6718 HT Ede, The Netherlands; J.M.deGoeij@uva.nl (J.M.d.G.); ronald.osinga@wur.nl (R.O.)
[2] Marine Animal Ecology, Wageningen University, P.O. Box 338, 6700 AH Wageningen, The Netherlands; tim.wijgerde@wur.nl
[3] Studio Associato Gaia, Piazza della Vittoria 15/23, 16121 Genova, Italy; a.sara@studioassociatogaia.com
[4] Department of Freshwater and Marine Ecology, Institute for Biodiversity and Ecosystem Dynamics, University of Amsterdam, P.O. Box 94248, 1090 GE Amsterdam, The Netherlands
* Correspondence: mert.gokalp@gmail.com; Tel.: +90-5377086534

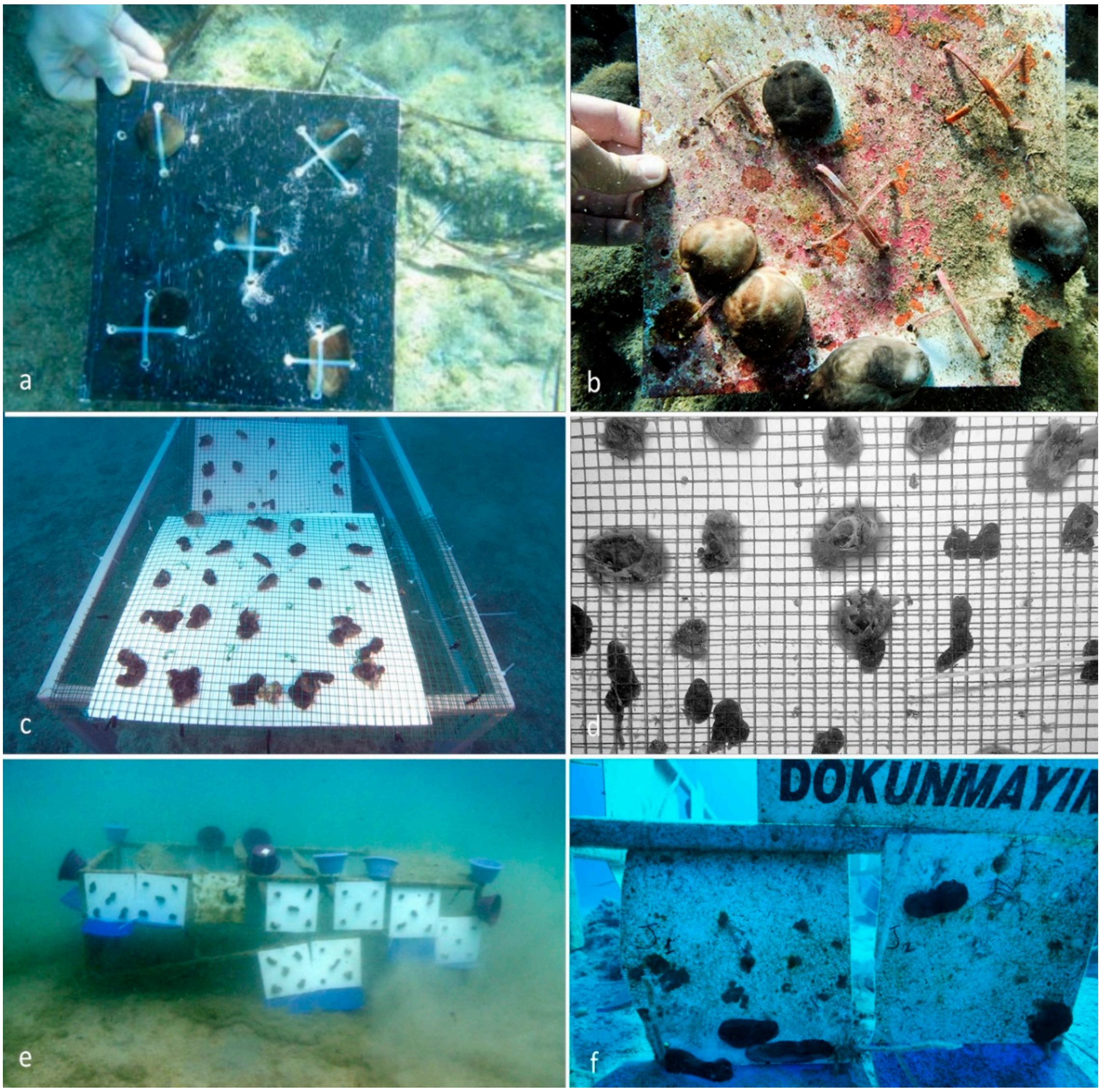

Figure S1. Overview of culture methods; (**a**) 1st trial; explants tie–wrapped to black PVC, experiment start (**b**) 1st trial; sponges tie–wrapped to white PVC, 1 year later explants splitting into two fragments and relocating position. (**c**) 2nd trial, polluted site; image of 50 × 50 cm plates, sponge explants secured with chicken wire (0–30° plates can be seen, 25 additional explants are on the other side of each plate). (**d**) 2nd trial, pristine site; infected explants shortly after seeding. (**e**) 3rd trial, polluted site; first upscaling of aquaculture of *Chondrosia reniformis* on vertical PVC plates. Explants are attached with gel–based polyacrylate superglue to PVC plates (10 plates per site). (**f**) 3rd trial, pristine site; explants after 4 months of culture. Some of the explants tend to travel towards the polypropylene plate and grow on it, but others prefer to stay on the PVC plates.

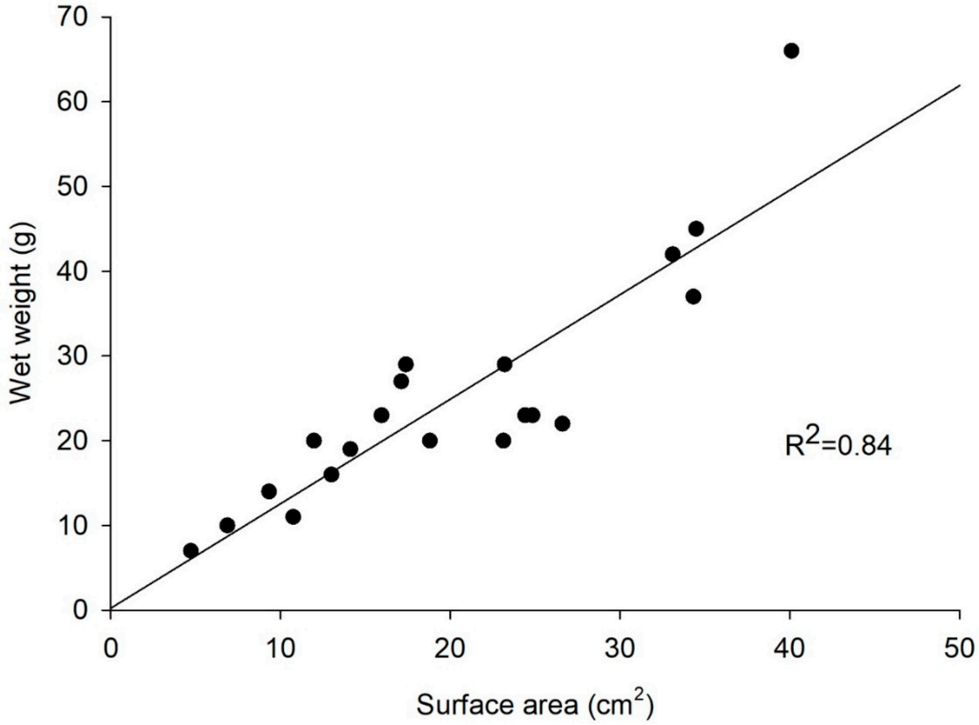

Figure S2. Correlation between surface area (cm^2) and wet weight (g) for *Chondrosia reniformis* (Pearson correlation, r = .92, n = 20, p = 0.000, two-tailed).

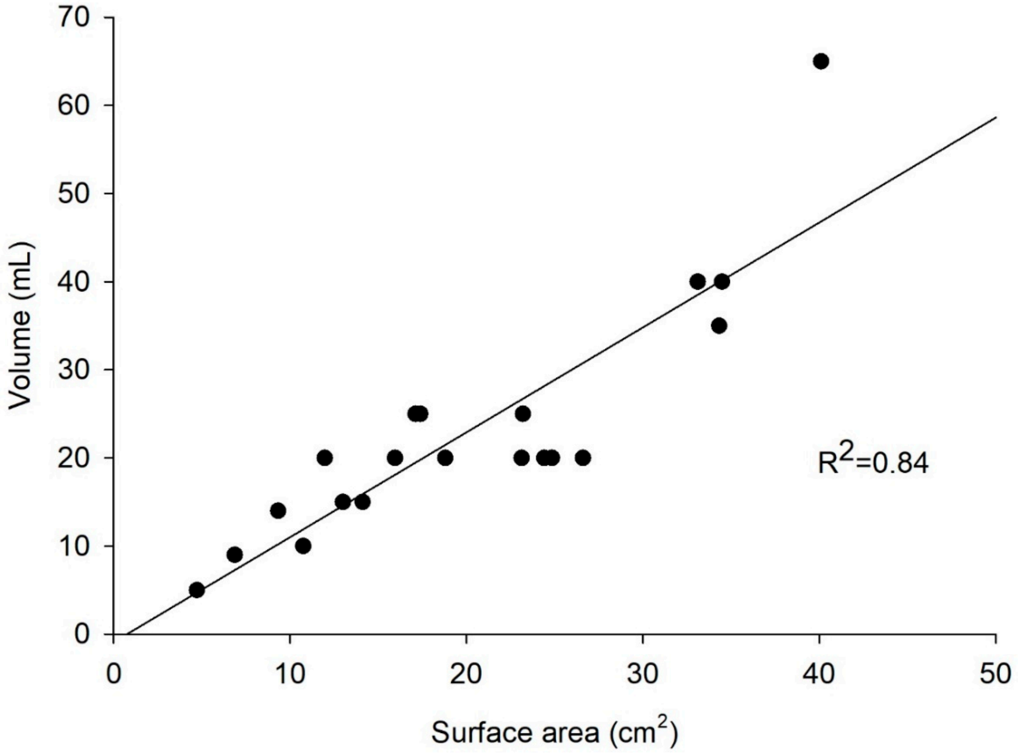

Figure S3. Correlation between surface area (cm^2) and volume (mL) for *Chondrosia reniformis* (Pearson correlation, r = .92, n = 20, p = 0.000, two-tailed).

Table S1. Experimental design of aquaculture trials 2 and 3. In Trial 3, 10 explants were taken per parent sponge, five explants were attached to each plate.

Trial	Sponge Species	Culture Site	PVC Plates	Explants per plate	Explants per angle	Total Explant Number
2	*Chondrosia reniformis*	Pristine	5	50	25	250
2	*Chondrosia reniformis*	Polluted	5	50	25	250
3	*Chondrosia reniformis*	Pristine	20	5		100
3		Polluted	20	5		100
